# Pleomorphic Pulmonary Manifestations of IgG4-Related Disease

**DOI:** 10.1155/2019/7572869

**Published:** 2019-08-20

**Authors:** Srikanth Naramala, Sharmi Biswas, Sreedhar Adapa, Vijay Gayam, Romeo C. Castillo, Srinadh Annangi, Venu Madhav Konala

**Affiliations:** ^1^Attending Rheumatologist, Department of Rheumatology, Adventist Medical Center, Hanford, CA, USA; ^2^Clinical Research Assistant, Weil Cornell Medicine, New York, NY, USA; ^3^Attending Nephrologist, The Nephrology Group, Fresno, CA, USA; ^4^Assistant Program Director, Department of Medicine, Interfaith Medical Center, Brooklyn, NY, USA; ^5^Program Director, Hanford Family Medicine Residency, Hanford, CA, USA; ^6^Fellow, Division of Pulmonary and Critical Care, University of Kentucky, Lexington, KY, USA; ^7^Medical Oncologist, Ashland Bellefonte Cancer Center, Ashland, KY, USA

## Abstract

Immunoglobulin G4-related disease (IgG4-RD) is a fibroinflammatory disorder which has been first reported in 2001 by Hamano and colleagues in a patient with autoimmune sclerosing pancreatitis. Almost every organ in the human body can be affected by IgG4-RD from infiltration with IgG4-positive plasma cells. Involvement of lungs with IgG4 is reported previously, but still, there is no clear picture of the pathophysiology behind lung involvement. Here, we are presenting a patient who has IgG4-RD presenting as pseudotumor of the lungs.

## 1. Introduction

The IgG4-related disease is characterized by increased serum IgG4 concentration along with pathologic findings of lymphoplasmacytic infiltration of IgG4-positive plasma cells, storiform fibrosis, and obliterative phlebitis in different organs. Some of the manifestations of IgG4-related disease include but not limited to autoimmune pancreatitis, tubulointerstitial nephritis, Mikulicz's disease, pseudotumor, aortitis, pericarditis, meningitis, Riedel's thyroiditis, and retroperitoneal fibrosis [1]. The IgG4-related disease often appears as a mass in the affected organ, for example, a renal mass, pseudotumor in orbit, and pulmonary nodular lesions. In the case of pulmonary IgG4-related disease, it is challenging to differentiate from malignant tumors due to the presentation as a pseudotumor from plasmacytic infiltration and fibrillization. A biopsy is required for the final diagnosis. Most patients with these pseudotumors respond to glucocorticoids [2].

We are reporting a patient who presented with lung nodule along with mediastinal lymphadenopathy. We confirmed the diagnosis with lung biopsy status post lower lobe resection.

## 2. Case Report

A 76-year-old African American male presented to the rheumatology clinic with severe fatigue, unintentional weight loss, memory loss, and ambulatory dysfunction secondary to generalized weakness. He lost 40 pounds in the last two years. Past medical history includes hypertension, hyperlipidemia, COPD, and diabetes mellitus. His medications were glipizide, metformin, bupropion, atorvastatin, aspirin, lisinopril, salbutamol, senna, and docusate. No pertinent family history. He stopped smoking 1½ years ago, with 120 pack-year smoking history, quit drinking three years ago, and smokes marijuana. He was a construction worker in the past. Physical examination was unremarkable except subjective weakness. Labs are significant for elevated creatinine level of 1.49 mg/dl (0.5–1 mg/dl), elevated IgE level of 234 (1–180 KU/L), proteinuria, and glucosuria.

Prior to this presentation, he was found to have worsening renal function with a creatinine level of 1.4 as an outpatient. An ultrasound image of Kidneys showed echogenic foci in the right kidney, which was further evaluated with CT (computerized tomography) scan of the abdomen and pelvis with contrast. It showed a scar in the interpolar region of the right kidney and also showed incidental findings of bronchiectasis and centrilobular emphysematous changes at lung bases. Later, CT scan of the chest with contrast was done which showed a similar finding and a 10 mm low-density area in the right thyroid lobe. Further evaluation by thyroid ultrasound showed bilateral thyroid nodules, including a 3.5 cm hypoechoic nodule in the lower pole. Fine-needle aspiration cytology of the nodule showed benign follicular nodule.

**Figure 1:**
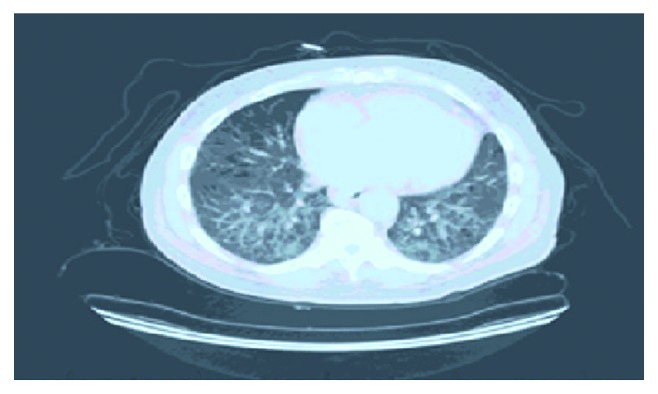
CT chest with contrast showing bilateral interlobular septal thickening, multiple ground-glass opacities, and cystic changes.

**Figure 2:**
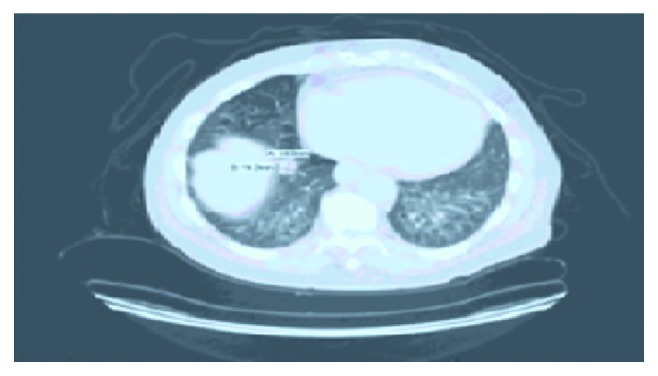
CT chest with contrast showing 1.4 cm right lower lobe lung nodule.

He had a repeat CT chest with contrast after 20 months concerning his progressively worsening weight loss and smoking history which showed 1.4 cm right lower lobe lung nodule, bilateral interlobular septal thickening with lower lobe predominance, multiple bilateral ground-glass opacities, cystic changes, and multiple enlarged mediastinal lymph nodes (Figures 1 and 2). PET (positron emission tomography) scanning was done which showed right lower lobe hypermetabolic nodule (SUV 6), highly suspicious for malignancy with extensive smoking history and significant weight loss.

**Figure 3:**
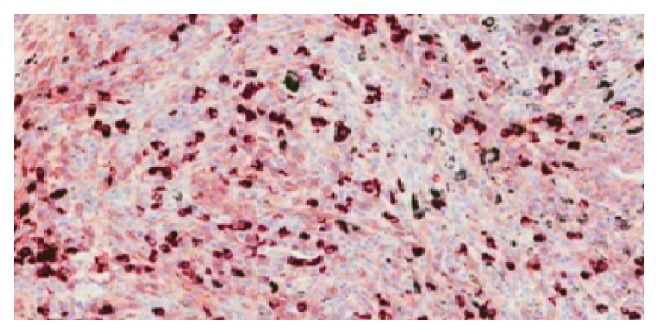
Immunostaining showing abundant IgG4 plasma cells.

**Figure 4:**
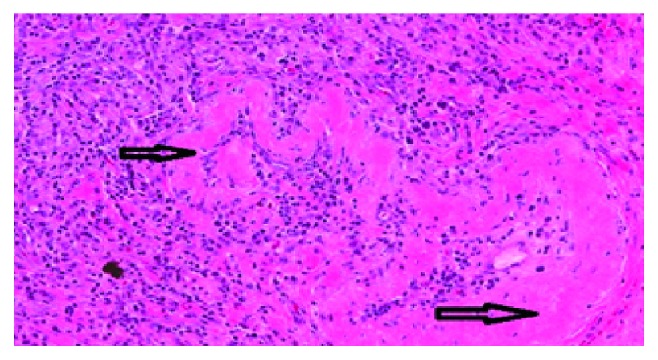
High power field showing obliterative phlebitis with destruction of the vessel wall.

**Figure 5:**
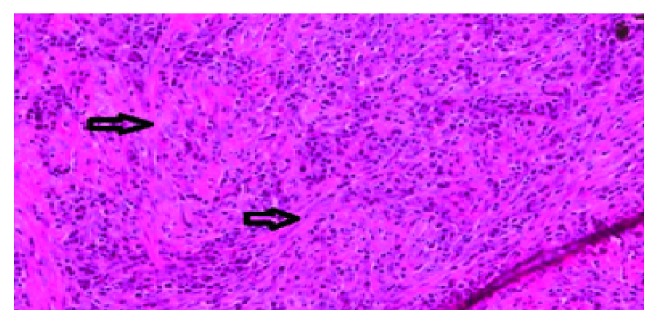
High power field showing fibrous bands.

**Figure 6:**
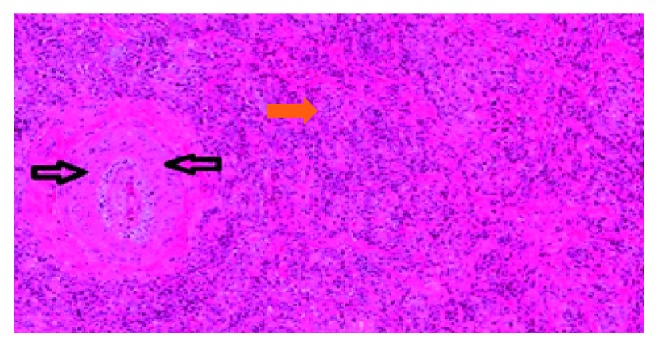
High power field showing arteritis with minimal obliteration of the lumen. The orange arrow shows rich lymphoplasmacytic infiltrate.

He underwent right lower lobe basilar segmentectomy and mediastinal lymph node dissection. Histology was negative for malignancy and showed numerous positive IgG4 plasma cells (more than 50 positive cells per higher power field) (Figure 3) along with obliterative phlebitis (Figure 4), significant fibrosis (Figure 5), and arteritis as lymphoplasmacytic infiltration (Figure 6). IgG4 : IgG ratio on the lung biopsy was greater than 40%. Lung biopsy was highly suggestive of IgG4-related disease. The lymph node also showed lymphoplasmacytic infiltration, IgG4-positive cells, irregular fibrous bands, but no obliterative phlebitis or arteritis.

He was started on 40 mg of prednisone, which resolved his fatigue and had significant weight gain, and his prednisone was tapered down and stopped in 4 months. He was not placed on any immunosuppression and was monitored for any further relapse. He is in remission for last one year off any medications without any relapses.

## 3. Discussion

IgG4-related disease (IgG4-RD) is a systemic fibroinflammatory disease with protean manifestations involving virtually any organ in the body. Hallmark of IgG4-related disease is lymphoplasmacytic tissue infiltration, fibrosis (often in the storiform pattern), obliterative phlebitis, and elevated serum IgG4 concentration. The disease is most commonly seen in middle-aged and older people (mean age from 59 to 68 years) and is more common in men (70 to 80 percent) [3, 4]. The incidence and prevalence of IgG4-related disease is still unknown, but now, it is considered as a part of a group of illness including Riedel's thyroiditis, Mikulicz's syndrome, Küttner's tumor, inflammatory pseudotumor, Ormond's disease, multifocal fibrosclerosis, and autoimmune pancreatitis.

Sarles et al. first described a form of IgG4-RD as sclerosing pancreatitis in 1961 [5]. It was later termed as autoimmune pancreatitis (AIP) by Yoshida et al. in 1995 [6]. Hamano et al. found elevated serum IgG4 levels in patients with autoimmune sclerosing pancreatitis among the Japanese population and described it as immunoglobulin G4-related disease (IgG4-RD) [7].

The pathophysiology of IgG4-related disease is still not well understood as it is a newly recognized disease. Autoimmunity is one of the commonest triggers for IgG4-related disease with a key role of T helper-2 (Th2) cell involvement in the pathophysiology. Th2 cells overexpress interleukins 4, 5, 10, and 13 as well as transforming growth factor *β* (TGF-*β*) which results in increased eosinophils as well elevated IgG4 and IgE as concentrations. It also contributes to the activation of fibroblasts leading to fibrosis [8].

Most of the patients with lung involvement in IgG4-RD are asymptomatic or with nonspecific symptoms like cough, fever, chest pain, and dyspnea [9]. There are three major diagnostic criteria for IgG4-RD: (1) diffuse/localized mass or swelling in single or multiple organs found on clinical examination; (2) raised IgG4 level >135 mg/dL; (3) histological findings of marked lymphoplasmacytic infiltration, storiform fibrosis, and obliterative phlebitis along with infiltration of IgG4-positive plasma cells. Obliterative arteritis is a more common finding in the lungs than in other organs. It is sporadic to found isolated IgG4-RD in lungs especially solitary pulmonary nodules [10].

IgG4 : IgG tissue plasma cell ratios of >40% and >50%, and >10 IgG4 plasma cells per HPF have been used for the diagnosis of IgG4-RD. Masaki et al. found ratios >40% in tissue plasma cells had a sensitivity and a specificity of 94.4% and 85.7%, respectively, in the diagnosis of IgG4-RD. It was also demonstrated that fibrotic tissue areas have decreased IgG4 plasma cells [11].

Radiological features of IgG4-related lung disease are variable. The following findings have been reported on CT scan of the chest—solid nodular density, ground-glass opacities, bronchiectasis featuring like interstitial lung disease, honeycombing simulating pulmonary fibrosis, and thickening of bronchovascular bundles and interlobular septa. In a single patient, multiple radiological features can be found [12]. Pleomorphic features in chest CT should raise the suspicion of IgG4-related lung disease. In our patient, a pulmonary nodule was found on CT and PET showed hypermetabolic lesion suspicious for malignancy. Sometimes, lung biopsy might be required to confirm the diagnosis of IgG4-RD [10]. In the present case, lung biopsy confirmed the diagnosis of IgG4-RD.

Systemic corticosteroids are the first-line treatment in IgG4-RD. Although spontaneous remission can occur, adequate treatment with steroids is highly recommended to prevent fibrosis. In IgG4-related lung disease, prednisone 30 mg or higher is prescribed daily for 1-2 weeks following by tapered dose. No randomized clinical trials have been conducted, and there are no formal treatment guidelines. Anecdotal data and multiple case reports mention the use of other immunosuppressive agents like methotrexate, mycophenolate mofetil, cyclophosphamide, and tacrolimus [13]. Biologic agents (rituximab) and surgery are also available options to treat depending on the symptoms and clinical findings. Despite initial response to corticosteroids, there is a chance for relapse in the long term. A higher level of serum IgG4 and diffuse involvement are considered common risk factors to cause a relapse [9]. IgG4-RD has been described in association with malignancies like lymphoma, pancreatic cancer, and lung cancer. However, it remains unclear whether IgG4-RD increases the risk of malignancy.

## 4. Conclusion

We report an unusual case of IgG4-related disease presenting with symptoms, signs, and radiological findings mimicking lung malignancy. It is imperative to differentiate both diseases as management as well as prognosis is quite different.
